# Commentary - Radiology in India: The Next Decade

**DOI:** 10.4103/0971-3026.41868

**Published:** 2008-08

**Authors:** Govindji R. Jankharia

**Affiliations:** Consultant Diagnostic Radiologist and Imageologist, Bhaveshwar Vihar, 383, SVP Road, Mumbai, India Email: govind@jankharia.com

For one who has been practicing radiology for the last 42 years and seen the evolution of radiology from single x-ray machines, with dark room processing and dripping films, to digital radiography and cross-sectional imaging techniques like USG, CT, and MRI, it is not difficult to predict how radiology will develop over the next 10 years and what will be the fate of practicing radiologists and the various imaging modalities.

Let us first talk about the practice of radiology in India and what could change in the next 10 years. Providers of radiology services can be divided into four broad groups.

1. Institutions managed and funded by the central government, state governments, or municipal corporations.
2. Private hospitals managed by big business corporations
3. Private diagnostic imaging centers managed by a single radiologist or a group of radiologists.
4. Small x-ray clinics managed by a single radiologist

The teaching and post-graduate institutions funded directly by the central government are fairly well managed and administered as access to funds is not very difficult and therefore these institutions have been able to keep pace with the advances in imaging technology. The radiologists trained in these institutions are well exposed to the art and science of imaging and they are dependable and good in their diagnostic acumen.

The teaching hospitals and medical colleges managed by the state government or municipal corporations, barring a few, are in pathetic conditions. Equipments are not regularly updated and most of them malfunction regularly, there is poor staffing, and the training given to the post-graduates is far from satisfactory. Many institutions have no full-time heads of department. These institutions suffer from lack of vision at all levels: the minister in charge, the head of the institution, and the head of the department.

The second group comprises the hospitals managed by big corporates or business houses. These hospitals, at present, are the leaders in the field of imaging as far as equipment is concerned and the diagnostic facilities are fairly good. These hospitals are well managed by professionals who have to ensure their profitable functioning. However, the middle-income group tends to stay away from these hospitals as services are expensive. Many of these hospitals are recognized for training by the National Board of Examinations, but the teaching is not very good in most of these hospitals, barring a few. These hospitals compete with each other and in the process obtain the latest equipment and devices.

The third group comprises the private diagnostic centers, which have been started by radiologists and sometimes funded by businessmen. In private practice, depending upon the energy, acumen, enthusiasm, and vision of the radiologists, many of these centers have the capacity to be trendsetters. Many imaging techniques and modalities that are popular today were pioneered by individual private diagnostic centers.

The fourth group is of the individual or single radiologists, running small clinics with x-ray facilities and sonography units. These small private clinics serve the imaging needs of family physicians and basic preliminary imaging is done by these centers. These clinics have done yeomen service to society in the past as they are patient-friendly, doctor-friendly, and easily accessible.

As far as the equipments and imaging modalities are concerned, teaching hospitals run by the state government and municipal corporations lag behind significantly. This is mainly due to the poor vision of the people at the helm of affairs. If they do not keep pace with the advances in technology, they face the risk of being derecognized by the Medical Council of India.

The next decade will be the decade of privatization. The Indian economy is bullish and more and more corporates are getting involved in the health care industry. This is good for the country. Small clinics or individual radiologists cannot afford the huge investments required to run full-fledged diagnostic centers and they will have no choice but to join the big groups or be a part of a corporate organization. The small centers will not be able to survive in big metropolises unless they upgrade to digital systems and have high-end sonography units. Though the small centers will continue do well in the small and medium-sized towns, I feel that if they were to get together under one roof and acquire all the basic imaging facilities, they would of tremendous service to society and will not be held back by economic constraints.

Let us now talk about individual imaging modalities and the expected changes.

## Conventional Radiology

Conventional radiology in India is in a very peculiar situation. Many centers and hospitals have embraced digital technology, predominantly with computerized radiography (CR). There are about 1350-1400 CR systems in the country at present. However many big hospitals, medical colleges, and more than 50% of the imaging centers still continue with the analog systems, mainly because of economic reasons. In a diverse country like ours, this disparity will continue to exist and will not change much over the next decade. Picture archiving and communication systems (PACS) will start as more and more centers acquire CR or digital radiography (DR) systems. Teleradiography will be useful if small centers in remote small towns also start using digital technology, but this will take a long time, as the priorities in our country are different.

In conventional radiology, 90% of the work is radiography of the chest, bones, spines, and sinuses and only 10% of the work is, and will be, related to procedures such as barium studies, intravenous urograms, etc.

## USG

This is the most popular cross-sectional imaging modality in India and it has served the poor as well as the rich equally well. There are about 50,000-55,000 USG machines in India and in the next 10 years, the number is going to double. For the radiologist running a small clinic, this is the most economically viable modality; charges to the patient vary from Rs. 150-2000, depending on the indications. The PNDT Act has crushed the misuse of USG services. Three- and four-dimensional imaging, USG-guided aspirations and biopsies, detection of fetal anomalies, and prenatal treatment of the fetus, are all possible with USG. Any center providing good USG services will prosper. It is, and will be, the first line of investigation for many ailments, as is the case with the chest x-ray.

## CT scan

There are about 3000 scanners throughout India and the potential of this modality is so high that in the next 10 years, I will not be surprised, if the number doubles. India has not lagged behind in acquiring the latest technologies in CT scan. Now, multidetector CT scanners are commonly seen in use and 64- and 128-slice scanners have become a reality. Indications for CT scans are increasing by the day.

## MRI

This is one of the most powerful imaging modalities in radiology, with the ability to provide superb soft tissue contrast; it is the imaging tool of the future. The revolution that it has brought about in medical imaging has been unparalleled. There are about 600 MRI scanners in India today; by the end of the next decade, the number is likely to have increased to 1000 or 1200. If the numbers are not very impressive, it is because of the high cost of the machine. However, with the economy booming and the GDP growth at 8-9%, it may become possible to acquire MRI equipment more easily and ensure that its benefits become available even to the poorest in the land. Functional MRI, spectroscopy, cardiac MRI, etc., will become routine studies over the next decade.

## PET (Positron Emission Tomography)

There are about 16 PET scanners in India and number is going to go up. Molecular imaging will be the main thrust throughout the world in the next decade and India is catching up. More and more PET/CT scanners are being installed in the major metros.

## Conclusion

In the next decade, conventional radiology will have a very limited role to play, being mainly restricted to chest x-rays and bedside radiography. USG facilities will double and CT scan and MRI will continue to grow in an exponential way, with more and more indications being included. Molecular imaging will become available in all the big metros. India will need about 20,000 radiologists if the benefits of imaging are to reach every corner of the country. Small x-ray clinics and diagnostic centers, if they want to survive and practice all the modalities, will have to start using digital technology and tie up with big groups.

